# Supplementary material for: Dissociation between the critical role of ClpB of Francisella tularensis for the heat shock response and the DnaK interaction and its important role for efficient type VI secretion and bacterial virulence
Source: PLoS Pathog. 2020 Apr 10;16(4):e1008466. doi: 10.1371/journal.ppat.1008466 (PMC7182274; doi:10.1371/journal.ppat.1008466)
Supplement: S1 Fig — (A) For the conserved score calculation, the query sequence of ClpB (Accession No:AJJ47289) was used as input in the ConSurf program. The boxes represent the residues of the Walker motifs and the important residues of Arginine fingers. WA1; Walker A motif of nucleotide binding domain (NBD)-1; WA2; Walker A motif of NBD-2; WB1; Walker B motif of NBD-1; WB2; Walker B motif of NBD-2; Arg-1: Arginine finger residue of NBD-1; and Arg-2: Arginine finger residue of NBD-2. (B) For the conserved score calculation, the query sequence of DnaK (Accession No: APC95585) was used as input in the ConSurf program. Conservation scale is from 1 to 9 and the color codes for the conservation are depicted in the figure. e—An exposed residue according to the neural-network algorithm. b—A buried residue according to the neural-network algorithm. f—A predicted functional residue (highly conserved and exposed). s—A predicted structural residue (highly conserved and buried). (PDF) [file ppat.1008466.s001.pdf]

S1A Fig: Evolutionary sequence conservation analysis of ClpB proteins

```
1          11         21         31         41
MNINKFTIKL QEALAEAQSY AFQQKATEFT SAHILKALLE QNDSVAISIL
eeeeebeeeb eebbeebebb eeeeeeeebb ebebbbbbbe eeeeebeebb
ff    f    f s   sfs  f            f       f
51         61         71         81         91
SVCGVNIQNF IKAVNDMVDS VAVLSGEGNP QVTPSRDLIA TLHKMQELAN
eebeeebeeb eeebeebbee beebeeeeee ebebbeebee bbebbeeebe
                                              f  ff  f
101        111        121        131        141
KNGDEFISSE VFLLASLEDK SLTGLYNKFG ITKEKLTKAV NDYRGGEKVS
eeeeebbbbb bbbbbbbeee ebeebbeeee eeeeebeebb eebeeeeeee
   f    ss                                     fff  f
151        161        171        181        191
SQNQEDMKGA LDKYTVDLTD LARKGKIDPI IGRDSEIRRT IQVLQRRTKN
eeeeeeeeeb beebbbebee ebeeeebeeb bbeeeebeeb bebbeeeeee
   ff  f   s f   f f   s  ffsffs ssff fsff   fssffffff
201     WA1211        221        231        241
NPVLIGEPGV GKTAIVEGLA QRIVNDEVPE GVKGKKVLSL DMGALLAGAK
eebbbbeeee bebbbbebbb eebbeeeeee ebeeeebbbb bbbbbbbbbe
ffs ss fff sfsss fsss ffs f fff      f  s s s   s sssf
251        261        271   WB1  281        291
FRGDFEERLK SVLKELSKQE GNVILFIDEL HTMVGAGKAE GSMDAGNMLK
beeebeeebe ebbeebbeee bebbbbbbeb bbbbbbbeee ebbebeebbe
 ff sfffsf  ss        s  s sssf  ss ssssf   f  fsffssf
301        311        321        331Arg1    341
PALARGELKC VGATTLDEYR EYVEKDPALE RRFQKVLVDE PTVEDTIAIL
ebbeebebbb bbbbbbeebe eebeeeeebe eebeebbeee eeeeebbbbb
fssffsfsss  sssss fsf    fffffsf ffsff  f f f ff s sss
351        361        371        381        391
RGLKERYELH HGVNITDSAI VSAATLSHRY ITDRQLPDKA IDLVDEAASQ
eebeeebeee ebbbbbebbb bbbbebeeeb beeeebeeeb bebbeebbbe
ffsff sf f f s ssf ss   ss sf fs s ff sfffs sfs ffs
401        411        421        431        441
IRMEIDSKPE KMESLYRRII QLKMQREQLK KEKDDATKKR LEILEQEIKG
bebebeeeee ebeebeeebb ebeeeeeebe eeeeeebeee beebeeebee
sf f fffff   f   ff s f fff ff   ff f     f
451        461        471        481        491
LDSEYKGLEE LWKAEKLKMQ GTSKLKEELE KAKFELEKYQ RVGDLSKMAE
eeeebeebee bbeeeeeeee eeeebeeebe ebeeebeebe eeeebeebbe
            s  ff     f    f                f        f
501        511        521        531        541
LQYGKIPELE AQIKQIEETE AEPSENKLVR TSVTENEIAD VVSKATGIPV
beeeebeebe eebeebeeee eeeeeeebbe eebeeeebbe bbbebeebeb
 fff  f sf                   s f   s   f s   s   ffsf
```

```
551        561        571        581        591
SKMMEGEKDK LLNMESFLHK RVIGQDQAIK AVSNAVRRSR SGLSDPNRPI
bebbeeeeee bbebeeebee ebbeeeebbe bbbebbeebe eebebeeeee
  s f f  f s  sf      s ff fs   s  ssffsf ffs sf  f 
601    WA2 611        621        631        641
GSFMFLGPTG VGKTELTKAL AEFLFDDEDA MLRVDMSEFM EKHSVARLIG
bbbbbbbeee eeeeebbebb bebbbebeeb bbbbbbbebb eeebbbebbe
sss sssfff fffffs f s s s sf       s sssf s fff s fs f
651        661        671    WB2 681        691
APPGYVGYEQ GGYLTEHVRR KPYSVILLDE VEKAHADVFN ILLQVLDDGR
bbeeebbeee eeebeebbee eebbbbbbee beeeeeebbe bbbebbeebe
ssfffssfff ff sff  ff  fsss s ff  ffff fssf  ssfssffsf
701        711        721        731        741
LTDGQGRTVD FKNTVIVMTS NLGSHRIQEM QGQDYDTVKD AVMEMVLSHF
beeeeeeeee bebbbbbbbb bbebebbeee eeeebeebee bbbebbeeeb
sfffffffff s s s  sss s fs  sf              s       s
751  Arg2  761        771        781        791
RPEFVNRVDD AIVFEPLNKE MITEIAKIQI KRLEKRLADL SIGLEVTTEA
eeebbeebee bbbbbbbeee ebeebbebeb eebeeebeee ebebebbeeb
fffs ff f   ssss s        s   f       f               
801        811        821        831
MDKLADAGFD PVFGARPLKR AIQNNLENPL ALKLLDGEFK
beebeeeebe ebbbbeebee bbeeebeeeb beebeeeeee
       f f f  ssffsff  sf   ff s     f f   
```

**Legend:**

**The conservation scale:**

| 1 | 2 | 3 | 4 | 5 | 6 | 7 | 8 | 9 |
|---|---|---|---|---|---|---|---|---|

**Variable** **Average** **Conserved**

**e - An exposed residue according to the neural-network algorithm.**

**b - A buried residue according to the neural-network algorithm.**

**f - A predicted functional residue (highly conserved and exposed).**

**s - A predicted structural residue (highly conserved and buried).**

**X - Insufficient data - the calculation for this site was performed on less than 10% of the sequences.**

S1B Fig: Evolutionary sequence conservation analysis of DnaK proteins

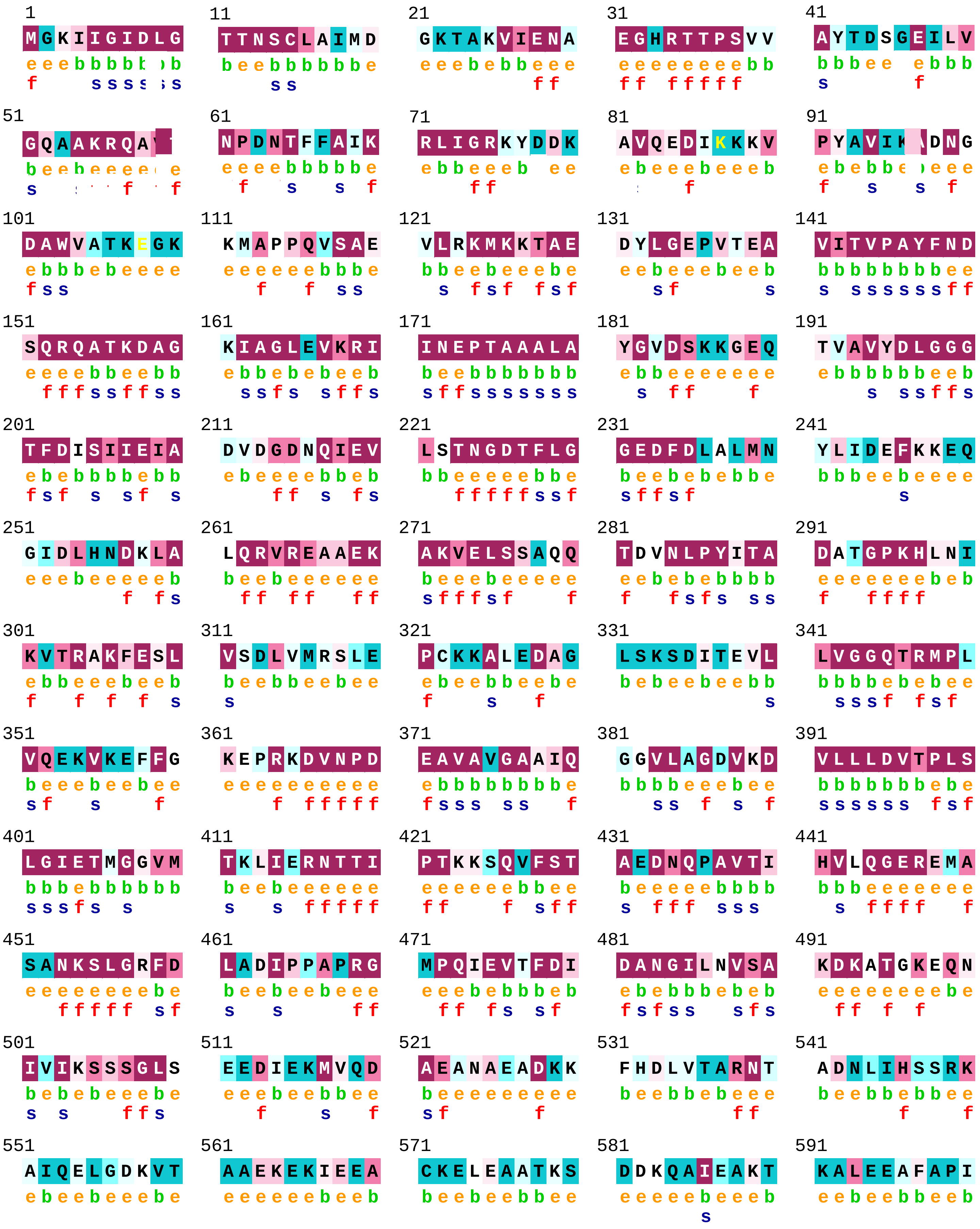

```
601                 611                 621                 631                 641
A Q K A Y A E Q A Q   A A G A Q G G A K A   E E P K K E E D V V   D A D F E D V E D D   K K
b e e b e e e e e e   e e e e e e e e e e   e e e e e e e e b b   e b e b e e b e e e   e e
                                                            s   f s f   f f s             f
```

**The conservation scale:**

| 1 | 2 | 3 | 4 | 5 | 6 | 7 | 8 | 9 |
|---|---|---|---|---|---|---|---|---|

**Variable** **Average** **Conserved**

- e - An exposed residue according to the neural-network algorithm.
- b - A buried residue according to the neural-network algorithm.
- f - A predicted functional residue (highly conserved and exposed).
- s - A predicted structural residue (highly conserved and buried).
- X - Insufficient data - the calculation for this site was performed on less than 10% of the sequences.
