# Supplementary figures and images for: Dissociation between the critical role of ClpB of Francisella tularensis for the heat shock response and the DnaK interaction and its important role for efficient type VI secretion and bacterial virulence
Source: PLoS Pathog. 2020 Apr 10;16(4):e1008466. doi: 10.1371/journal.ppat.1008466 (PMC7182274; doi:10.1371/journal.ppat.1008466)

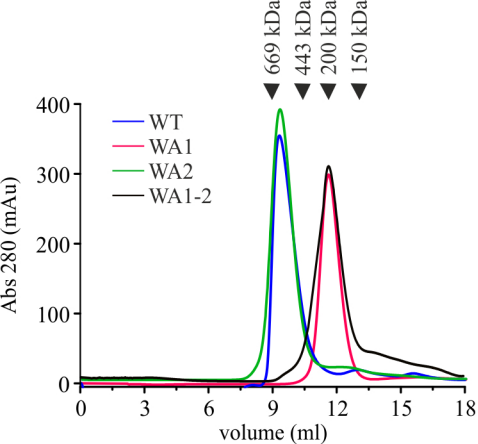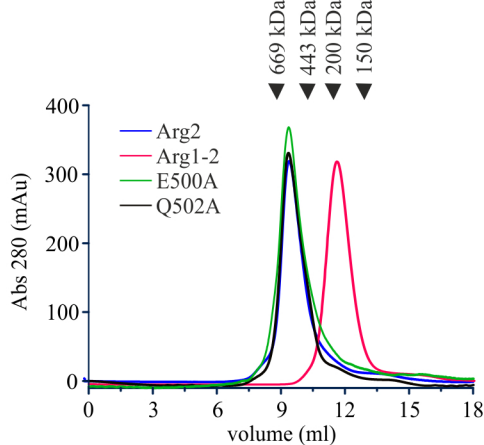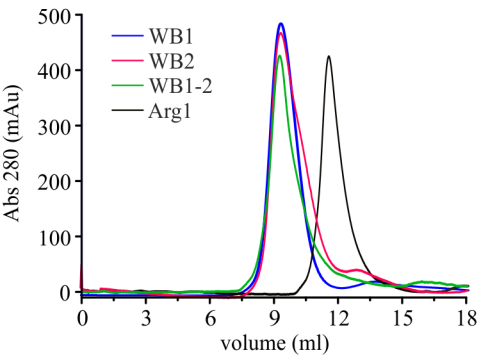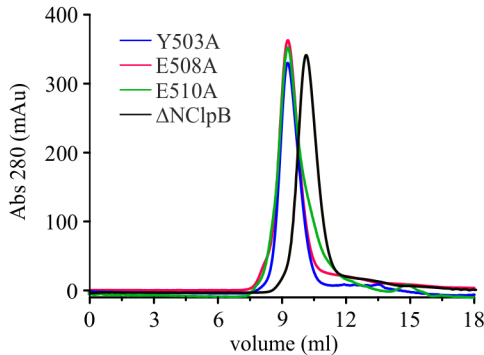

Supplement: S2 Fig — Elution profile of ClpB, or the ClpB variants were determined in the presence of 2 mM ATP in running buffer as described in Materials and Methods. Elution profiles of four proteins in each sub-figures are placed together for better visibility. Molecular size standards used were Thyroglobulin (669 kDa), Apoferritin (443 kDa), Amylase (200 kDa), and Alcohol Dehydrogenase (150 kDa) and their positions are indicated. (PDF) [file ppat.1008466.s002.pdf]

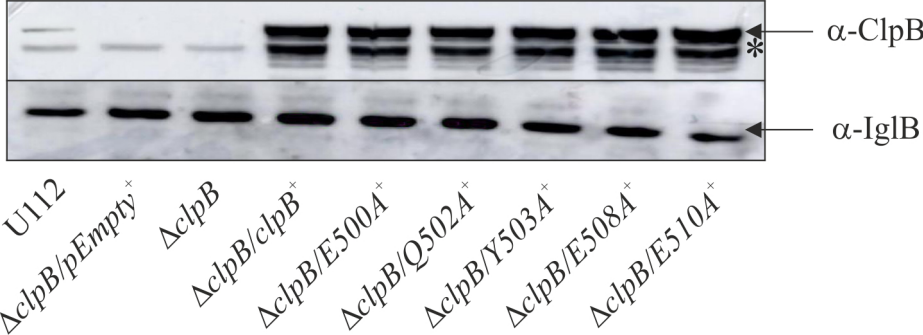

Supplement: S3 Fig — Whole cell lysate of wild type, ΔclpB or M-domain variants complemented in trans in ΔclpB were prepared, separated by SDS-PAGE and probed with ClpB antibody from Synechocytis PCC 6803 (slr1642, Agrisera) that cross reacts with the Francisella ClpB. Anti-IglB was used as a loading control. Vector indicates the empty vector (pKK289). Asterisks indicate non-specific bands. Assays were repeated at least twice and a representative blot is shown. (PDF) [file ppat.1008466.s003.pdf]

A

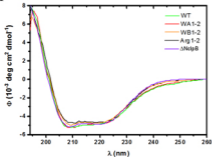

B

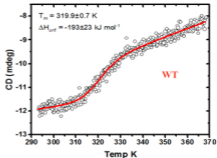

C

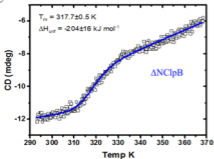

D

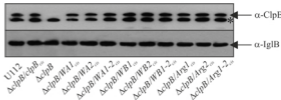

Supplement: S5 Fig — (A) Far-UV CD spectra of the purified wild type ClpB and the mutant variants recorded between 200 nm– 250 nm at 25°C. The protein concentration was 10 μM in 10 mM NaPi, 30 mM NaCl, at pH 7.5. The CD signal was expressed as the mean molar residue ellipticity. (B-C) Temperature-induced changes in the CD signal at 220 nm of WT and ΔN ClpB were recorded between 20 and 75°C using a scan rate of 0.5 °C / min. The solid line shows the fit of a two-state unfolding model. (D) Western immunoblot analysis of the total cell lysates from the indicated strains probed with ClpB antibody from Synechocytis PCC 6803 (slr1642, Agrisera) that cross reacts with the Francisella ClpB. IglB was used as loading control. Asterisks indicate non-specific bands. Assays were repeated at least twice and representative blots are shown. (PDF) [file ppat.1008466.s005.pdf]

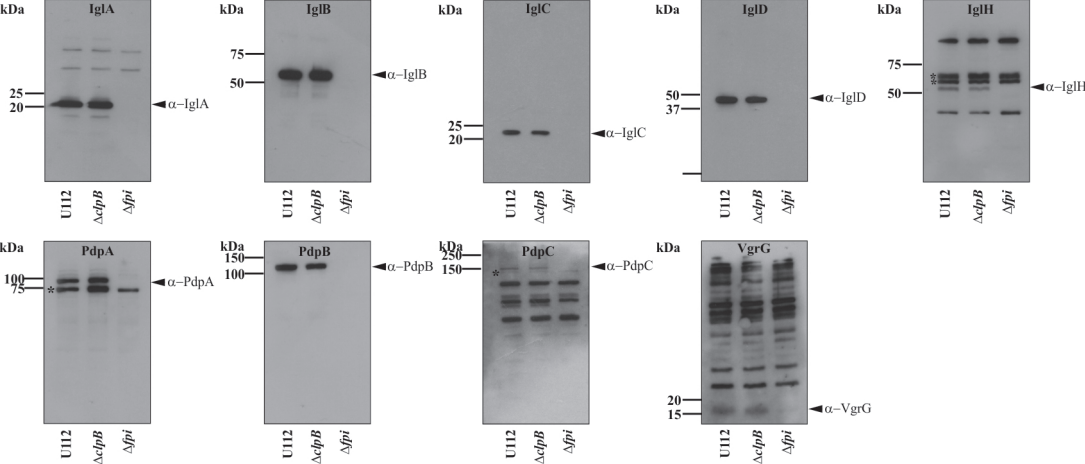

Supplement: S6 Fig — Whole cell lysate of each strain of ClpB variants and wild type was prepared, separated by SDS-PAGE and probed with specific antibodies against indicated FPI proteins. U112: F. novicida wild type, ΔclpB: clpB-deleted strain, and Δfpi: Francisella Pathogenicity Island (FPI)-deleted F. novicida strain. Details about the antibodies used are described in Materials and Methods. Asterisks indicate non-specific bands. Assays were repeated at least twice and representative blots are shown. (PDF) [file ppat.1008466.s006.pdf]

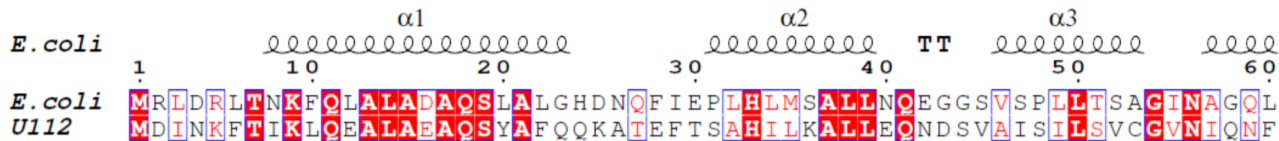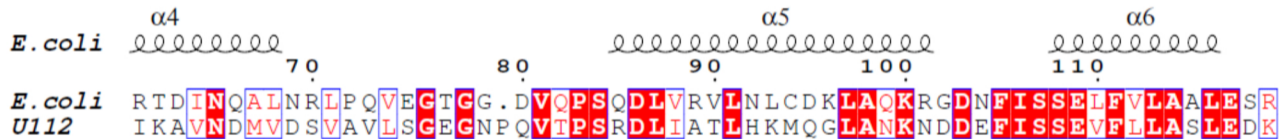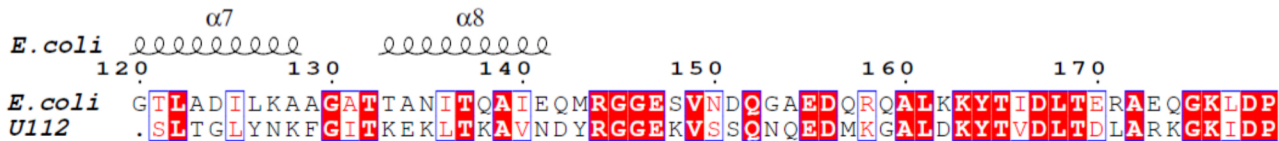

Supplement: S7 Fig — ClpB sequences of E. coli and F. novicida U112 were retrieved from NCBI (https://www.ncbi.nlm.nih.gov/), sequence alignments were performed using MAFFT (https://mafft.cbrc.jp/alignment/server/), and the corresponding image was generated using the web server ESPript 3 (http://espript.ibcp.fr). The first 180 of N-terminal domain (1–156 aa) of the E. coli-U112 ClpB alignment is shown. Secondary structure elements as predicted for E. coli ClpB are displayed above the alignment. (PDF) [file ppat.1008466.s007.pdf]
