# Supplementary material for: Dissociation between the critical role of ClpB of Francisella tularensis for the heat shock response and the DnaK interaction and its important role for efficient type VI secretion and bacterial virulence
Source: PLoS Pathog. 2020 Apr 10;16(4):e1008466. doi: 10.1371/journal.ppat.1008466 (PMC7182274; doi:10.1371/journal.ppat.1008466)
Supplement: S4 Fig — ClpB sequences was retrieved from the NCBI server (https://www.ncbi.nlm.nih.gov/). Sequence alignments of V. cholerae (Accession: AKB07899.1), F. novicida U112 (Accession: AJI61375.1), F. tularensis LVS (Accession: CAJ78535.1), F. tularensis SCHU S4 (Accession: YP_170660.1), E. coli (Accession: BAA16476.1), Mycobacterium tuberculosis (Accession: LB17500.1), and Pseudomonas aeruginosa (Accession: WP_058169183.1) were performed using MAFFT (https://mafft.cbrc.jp/alignment/server/) and the corresponding image generated using the server ESPript 3 (http://espript.ibcp.fr). Secondary structure elements based on the V. cholerae ClpB crystal structure are displayed above the alignment. Start and end of the N-terminal domain is marked with brown arrows. Conserved Walker A (206-GX4GKT-213 and 605-GX4GKT-612) and Walker B (276-Hy2DE-279 and 675-Hy2DE-678) motifs, where X = any amino (aa) and Hy = hydrophobic amino acids are indicated with blue boxes. Conserved Arg residues (R) present in both nucleotide binding domains (Arg-332 and Arg-756) are proposed to serve as Arg fingers and indicated with a blue box, The conserved residue of each of these motifs (highlighted in yellow) was subjected to alanine mutagenesis. (PDF) [file ppat.1008466.s004.pdf]

# S4 Fig: Sequence alignment of ClpB proteins

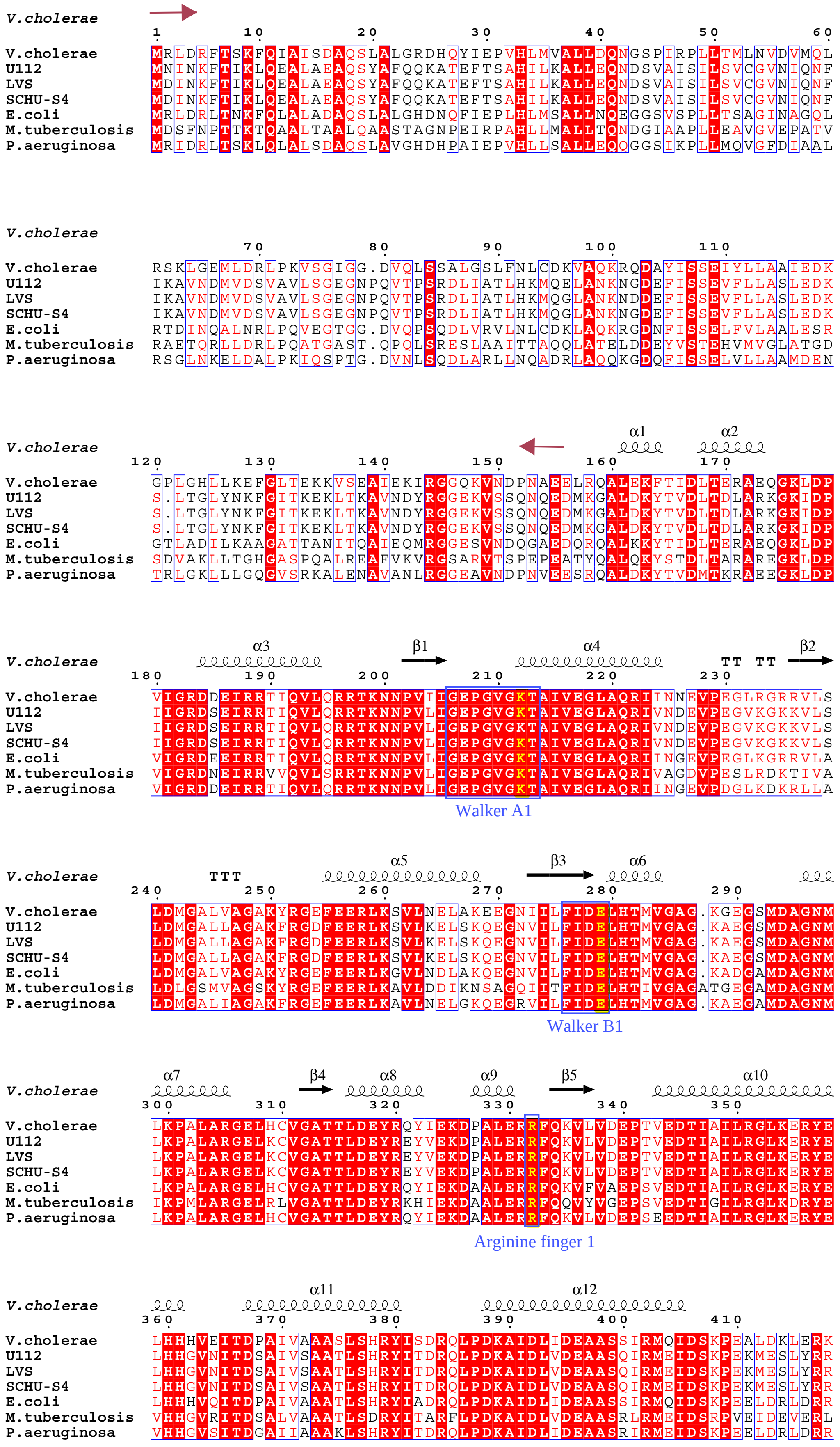

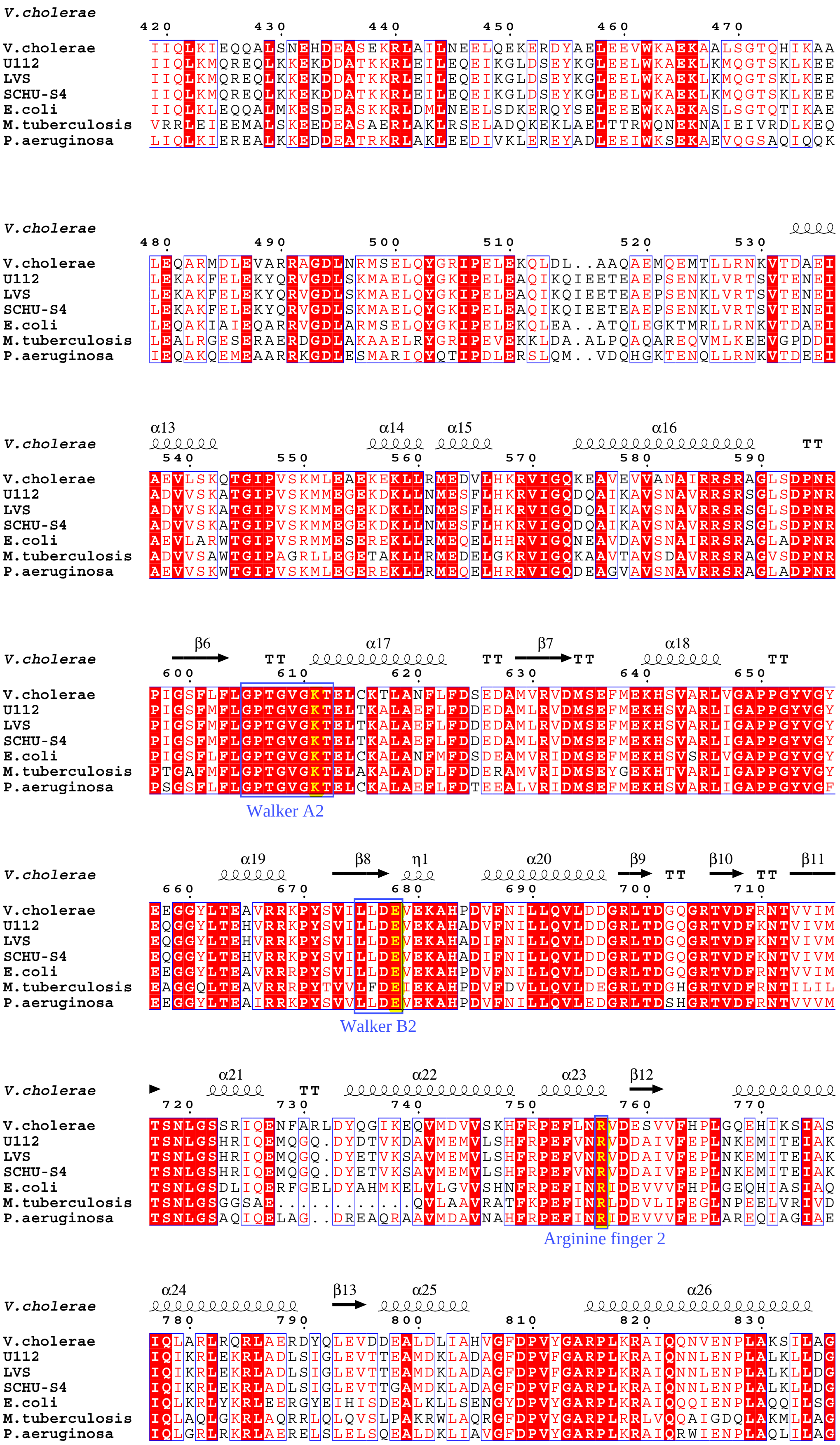
V.cholerae
U112
LVS
SCHU-S4
E.coli
M.tuberculosis
P.aeruginosa
Walker A2
Walker B2
Arginine finger 2

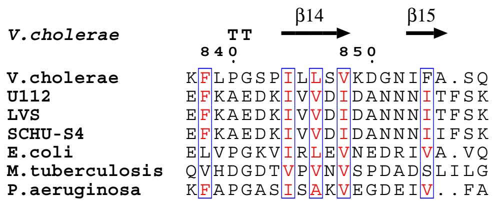
β14
β15
V.cholerae
TT
840
850
V.cholerae KFLPGSPILLSVKDGNIFA.SQ
U112 EFKAEDKIVVDIDANNNITFSK
LVS EFKAEDKIVVDIDANNNITFSK
SCHU-S4 EFKAEDKIVVDIDANNNIIFSK
E.coli ELVPGKVIRLEVNEDRIVA.VQ
M.tuberculosis QVHDGDTVPVNVSPDADSLILG
P.aeruginosa KFAPGASISAKVEGDEIV..FA
