# Supplementary material for: Dissociation between the critical role of ClpB of Francisella tularensis for the heat shock response and the DnaK interaction and its important role for efficient type VI secretion and bacterial virulence
Source: PLoS Pathog. 2020 Apr 10;16(4):e1008466. doi: 10.1371/journal.ppat.1008466 (PMC7182274; doi:10.1371/journal.ppat.1008466)
Supplement: S1 Table — (DOCX) [file ppat.1008466.s008.docx]

**S1 Table. Strains and plasmids used in this study**

| Strain or plasmid | Relevant genotype and/or phenotype | Source or reference |
| --- | --- | --- |
| *E. coli* strains |  |  |
| TOP 10 | _F_^_^ *mcrA* Δ(*mrr-hsdRMS-mcrBC*) φ80*lacZ*ΔM15 Δ*lacX74 recA1 deoR araD139* Δ(*ara-leu*)*7679 galU* *galK rpsL* (Str^r^ ) *endA1 nupG* | Invitrogen |
| S17-1 λ*pir* | *recA thi pro hsdRM*^+^ Sm^r^ <RP4:2-Tc:Mu:Ku:Tn7> Tp^r^ | [[1](#_ENREF_1)] |
| *F. tularensis* strains |  |  |
| U112 | *F. novicida*, wild type | ATCC^a^ |
| U112 *ΔclpB* | *F. Novicida*, in-frame deletion of *clpB* | This study |
| U112 *ΔiglE* | *F. Novicida*, in-frame deletion of *iglE* | [[2](#_ENREF_2)] |
| U112 vgrG::Tn | *F. Novicida*, transposon mutant of *vgrG* | [[3](#_ENREF_3)] |
| U112 *Δfpi* | *F. novicida*, in-frame deletion of *Francisella Pathogenicity Island (fpi)* | [[4](#_ENREF_4)] |
| *In cis* complemented strains |  |  |
| *clpB_cis_* | U112 *ΔclpB,* complemented *in cis* with wild type *clpB* | This study |
| *WA1* | U112 *ΔclpB,* complemented *in cis* with *clpB* substituted with K212A in Walker A motif of NBD-1. | This study |
| *WA2* | U112 *ΔclpB,* complemented *in cis* with *clpB* substituted with K613A in Walker A motif of NBD-2. | This study |
| *WA1-2* | U112 *ΔclpB,* complemented *in cis* with *clpB* substituted with K212A and K613A in Walker A motif of NBD-1 and NBD-2 respectively. | This study |
| *WB1* | U112 *ΔclpB,* complemented *in cis* with *clpB* substituted with E279A in Walker B motif of NBD-1. | This study |
| *WB2* | U112 *ΔclpB,* complemented *in cis* with *clpB* substituted with E680A in Walker B motif of NBD-2. | This study |
| *WB1-2* | U112 *ΔclpB,* complemented *in cis* with *clpB* substituted with E279A and E680A in Walker B motif of NBD-1 and NBD-2 respectively. | This study |
| *Arg1* | U112 *ΔclpB,* complemented *in cis* with *clpB* substituted with R332A in Arginine finger motif of NBD-1. | This study |
| *Arg2* | U112 *ΔclpB,* complemented *in cis* with *clpB* substituted with R757A in Arginine finger motif of NBD-2. | This study |
| *Arg1-2* | U112 *ΔclpB,* complemented *in cis* with *clpB* substituted with R332A and R757A in Arginine finger motif of NBD-1 and NBD-2 respectively. | This study |
| *Y503A* | U112 *ΔclpB,* complemented *in cis* with Y503A substituted *clpB*. | This study |
| Plasmids |  |  |
| pCR-Blunt II-TOPO | Blunt end cloning vector, Km^r^ | Invitrogen |
| pDMK3 | Suicide plasmid carrying sacB, Km^R^ | [[5](#_ENREF_5)] |
| pALA012 | pDMK3 with wild type *clpB*, Km^r^ | This study |
| pALA013 | pDMK3 with wild type *clpB* flanked with upstream and downstream sequence, Km^r^ | This study |
| pALA014 | pALA013 with K212A mutation, Km^r^ | This study |
| pALA015 | pALA013 with E279A mutation, Km^r^ | This study |
| pALA016 | pALA013 with R332A mutation, Km^r^ | This study |
| pALA017 | pALA013 with K613A mutation, Km^r^ | This study |
| pALA018 | pALA013 with E680A mutation, Km^r^ | This study |
| pALA019 | pALA013 with R757A mutation, Km^r^ | This study |
| pALA020 | pALA013 with K212A/K613A mutation, Km^r^ | This study |
| pALA021 | pALA013 with E279A/E680A mutation, Km^r^ | This study |
| pALA022 | pALA013 with R332A/R757A mutation, Km^r^ | This study |
| pALA023 | pALA013 with Y503A mutation, Km^r^ | This study |
| pET-His1a | Modified pET expression vector, obtained from protein expression and purification facility, Umea university, Sweden | This study |
| pALA024 | pET-His1a with wild type clpB (codon optimized for *E. coli*), Km^r^ | This study |
| pALA025 | pALA024 with E500A mutation, Km^r^ | This study |
| pALA026 | pALA024 with Q5002A mutation, Km^r^ | This study |
| pALA027 | pALA024 with Y503A mutation, Km^r^ | This study |
| pALA028 | pALA024 with E508A mutation, Km^r^ | This study |
| pALA029 | pALA024 with E510A mutation, Km^r^ | This study |
| pALA043 | pALA024 with K212A mutation, Km^r^ | This study |
| pALA044 | pALA024 with E279A mutation, Km^r^ | This study |
| pALA045 | pALA024 with R332A mutation, Km^r^ | This study |
| pALA046 | pALA024 with K613A mutation, Km^r^ | This study |
| pALA047 | pALA024 with E680A mutation, Km^r^ | This study |
| pALA043 | pALA024 with R757A mutation, Km^r^ | This study |
| pALA043 | pALA024 with K212A/K613A mutation, Km^r^ | This study |
| pALA043 | pALA024 with E279A/E680A mutation, Km^r^ | This study |
| pALA043 | pALA024 with R332A/R757A mutation, Km^r^ | This study |
| pALA043 | pALA024 with N-terminal (2-156 aa) deletion mutation, Km^r^ | This study |
| pALA030 | pET-His1a with wilt type dnaJ, Km^r^ | This study |
| pALA031 | pET-His1a with wilt type dnaK, Km^r^ | This study |
| pALA032 | pET-His1a with wilt type grpE, Km^r^ | This study |
| pKK289-Km | Expression plasmid with LVS GroESL promoter, Km^r^ | [[6](#_ENREF_6)] |
| pALA033 | pKK289-Km with wild type clpB | This study |
| pALA034 | pALA033 with E500A mutation, Km^r^ | This study |
| pALA035 | pALA033 with Q5002A mutation, Km^r^ | This study |
| pALA036 | pALA033 with Y503A mutation, Km^r^ | This study |
| pALA037 | pALA033 with E508A mutation, Km^r^ | This study |
| pALA038 | pALA033 with E510A mutation, Km^r^ | This study |
| pALA039 | pALA033 with N-terminal (2-156 aa) deletion mutation, Km^r^ | This study |
| pALA040 | pKK289-Km with N-terminal (2-156 aa) codon optimized (for *Francisella*) *E. coli* clpB, fused with ∆N (157- 857 aa) *clpB* the *F. novicida* *U112*, Km^r^ | This study |
| pALA041 | pKK289-Km with wild type *E. coli str. K-12 substr. MG1655*h clpB, Km^r^ | This study |
|  |  |  |

^a^  Obtained from the American Type Culture Collection, Manassas, VA, USA,

1. Simon R, Priefer U, Pühler A (1983) A Broad Host Range Mobilization System for In Vivo Genetic Engineering: Transposon Mutagenesis in Gram Negative Bacteria. Bio/Technology 1: 784.

2. Bröms JE, Meyer L, Sjöstedt A (2017) A mutagenesis-based approach identifies amino acids in the N-terminal part of *Francisella tularensis* IglE that critically control Type VI system-mediated secretion. Virulence 8: 821-847.

3. Gallagher LA, Ramage E, Jacobs MA, Kaul R, Brittnacher M, et al. (2007) A comprehensive transposon mutant library of *Francisella novicida*, a bioweapon surrogate. Proc Natl Acad Sci U S A 104: 1009-1014.

4. Rigard M, Bröms JE, Mosnier A, Hologne M, Martin A, et al. (2016) *Francisella tularensis* IglG belongs to a novel family of PAAR-like T6SS proteins and harbors a unique N-terminal extension required for virulence. PLoS Pathog 12: e1005821.

5. Lindgren M, Bröms JE, Meyer L, Golovliov I, Sjöstedt A (2013) The *Francisella tularensis* LVS Δ*pdpC* mutant exhibits a unique phenotype during intracellular infection. BMC Microbiol 13: 20.

6. Bönquist L, Lindgren H, Golovliov I, Guina T, Sjöstedt A (2008) MglA and Igl proteins contribute to the modulation of *Francisella tularensis* live vaccine strain-containing phagosomes in murine macrophages. Infect Immun 76: 3502-3510.
