# Supplementary material for: Dissociation between the critical role of ClpB of Francisella tularensis for the heat shock response and the DnaK interaction and its important role for efficient type VI secretion and bacterial virulence
Source: PLoS Pathog. 2020 Apr 10;16(4):e1008466. doi: 10.1371/journal.ppat.1008466 (PMC7182274; doi:10.1371/journal.ppat.1008466)
Supplement: S2 Table — (DOCX) [file ppat.1008466.s009.docx]

**S2 Table. Oligonucleotides used in the study.**

|  | Oligonucleotides |
| --- | --- |
| U112 null mutants |  |
| *ΔclpB* | ClpB_5SacI (5’- G CCG AGC TCA GCC TCA TCT TCT ATC TCT-3’); ClpB_Int_R (5’-gcc tca tct aga gct tct tgt a-3’); ClpB_Int_F (5’- ctc tgg ctc taa aac ttc tag at-3’); ClpB­_3SalI (5’-acg cgt cga ccc ttg cct aaa gtt ata ggc t-3’); ClpB­_OF (5’- cca gtc ttc atc tat atc aaa t-3’) and ClpB­_OR (5’- ctg aag cat ctg agc tta aaa -3’) |
| Cloning into pET-His1a vector |  |
| pET-His1a-clpB_WT_ | ClpBopt_c876t_F (5'-ata tta cct gca tcc ata gaa ccc tcg gct tta cc-3') and ClpBopt_c876t_R (5'-ggt aaa gcc gag ggt tct atg gat gca ggt aat at-3'); ClpBopt_5NcoI(5'-GG CCA TGG ATA TCA ATA AAT TCA CG-3') and ClpBopt_3XhoI (5'-CCG CTC GAG TTA TTT GCT GAA GAT AAT GTT G-3') |
| pET-His1a-ΔNclpB | ClpBopt_del_1-156_F (5'-GTG TAT TTA TCT AAT GCA CCT TTC ATT ATT TCC CCT CAA GAA AAA TTA TCT GTT A-3') and ClpBopt_del_1-156_R (5'-TAA CAG ATA ATT TTT CTT GAG GGG AAA TAA TGA AAG GTG CAT TAG ATA AAT ACA C-3') |
| pET-His1a-clpB_E500A_ | ClpBopt_E500A_F (5'-ttt gcc ata ctg cag cgc ggc cat ttt gct cag-3') and ClpBopt_E500A_R (5'-ctg agc aaa atg gcc gcg ctg cag tat ggc aaa-3') |
| pET-His1a-clpB_Q502A_ | ClpBopt_Q502A_F (5'-cgg gaa ttt tgc cat acg cca gct cgg cca ttt tgc-3') and ClpBopt_Q502A_R (5'-gca aaa tgg ccg agc tgg cgt atg gca aaa ttc ccg-3') |
| pET-His1a-clpB_Y503A_ | ClpBopt_Y503A_F (5'-cag ttc ggg aat ttt gcc agc ctg cag ctc ggc cat ttt g-3') and ClpBopt_Y503A_R (5'-caa aat ggc cga gct gca ggc tgg caa aat tcc cga act g-3') |
| pET-His1a-clpB_E508A_ | ClpBopt_E508A_F (5'-tga tct gtg cct cca gtg cgg gaa ttt tgc cat ac-3') and ClpBopt_E508A_R (5'-gta tgg caa aat tcc cgc act gga ggc aca gat ca-3') |
| pET-His1a-clpB_E510A_ | ClpBopt_E510A_F (5'-att tgt ttg atc tgt gcc gcc agt tcg gga att ttg c-3') and ClpBopt_E510A_R (5'-gca aaa ttc ccg aac tgg cgg cac aga tca aac aaa t-3') |
| pET-His1a-dnaJ | DnaJopt_C549T-F (5'-tgc cct gac cat gac acg cat ggc agg-3') and DnaJopt_C549T-R (5'-cct gcc atg cgt gtc atg gtc agg gca-3'); DnaJopt_5NcoI (5'-GG CCA TGG TTT ACC CGC ACT TCA GC-3') and DnaJopt_3XhoI (5'-CCG CTC GAG TTA TTC AAA GAA TTT CTT CGC AT-3') |
| pET-His1a-dnaK | DnaKopt_c1215t_F (5'-cat cac acc gcc cat agt ctc aat acc cag g-3') and DnaKopt_c1215t_R (5'-cct ggg tat tga gac tat ggg cgg tgt gat g-3'); DnaKopt_5NcoI (5'-GG CCA TGG GCA AGA TCA TTG GTA TCG-3') and DnaKopt_3XhoI (5'-CCG CTC GAG TTA CTT TTT ATC GTC TTC AAC GTC C-3') |
| pET-His1a-grpE | GrpEopt_5NcoI (5'-GG CCA TGG CAA GCA GGA GAA AAG C-3') and GrpEopt_3XhoI (5'-CCG CTC GAG TTA GTT TTT AAC AAT AAC CAC CTT CG-3') |
| pET-His1a-WA1 | ClpBopt_K212A_F (5'-tcc aca atc gca gtc gcg cca acg ccc ggt tc-3') and ClpBopt_K212A_R (5'-gaa ccg ggc gtt ggc gcg act gcg att gtg ga-3') |
| pET-His1a-WA2 | ClpBopt_K613A_F (5'-ggg tcc gac tgg tgt tgg cgc aac aga att aac caa agc g-3') and ClpBopt_K613A_R (5'-cgc ttt ggt taa ttc tgt tgc gcc aac acc agt cgg acc c-3') |
| pET-His1a-WA1-2 | ClpBopt_K212A_F, ClpBopt_K212A_R, ClpBopt_K613A_F and ClpBopt_K613A_R |
| pET-His1a-WB1 | ClpBopt_E680A_F (5'-gtc att ctc ctt gac gcg gtg gag aaa gct cac-3') and ClpBopt_E680A_R (5'-gtg agc ttt ctc cac cgc gtc aag gag aat gac-3') |
| pET-His1a-WB2 | ClpBopt_E279A_F (5'-tca tct tgt tca ttg acg cac tcc ata cta tgg tgg g-3') and ClpBopt_E279A_ R (5'-ccc acc ata gta tgg agt gcg tca atg aac aag atg a-3') |
| pET-His1a-WB1-2 | ClpBopt_E680A_F, ClpBopt_E680A_R, ClpBopt_E279A_F and ClpBopt_E279A_ R |
| pET-His1a-Arg1 | ClpBopt_R332A_F (5'-ctg cgc tgg aac gtg cct tcc aga agg tgc-3') and ClpBopt_R332A_R (5'-gca cct tct gga agg cac gtt cca gcg cag-3') |
| pET-His1a-Arg2 | ClpBopt_R757A_F (5'-cgc cca gaa ttt gta aac gcc gtt gat gat gcg atc gt-3') and ClpBopt_R757A_R (5'-acg atc gca tca tca acg gcg ttt aca aat tct ggg cg-3') |
| pET-His1a-Arg1-2 | ClpBopt_R332A_F, ClpBopt_R332A_R, ClpBopt_R757A_F and ClpBopt_R757A_R |
| Cloning into pKK289km vector |  |
| pKK289-ΔNclpB | ClpB_del_2-156_F (5'-cta atg cac ctt tca tat gta tat ctc ctt ctt aaa tct gca gtg c-3') and ClpB_del_2-156_R (5'-gca ctg cag att taa gaa gga gat ata cat atg aaa ggt gca tta g-3') |
| pKK289-clpB_E500A_ | ClpB_E500A_F (5'-ggt gat ttg agc aaa atg gca gca tta caa tac ggt aaa ata cct-3') and ClpB_E500A_R (5'-agg tat ttt acc gta ttg taa tgc tgc cat ttt gct caa atc acc-3') |
| pKK289-clpB_Q502A_ | ClpB_Q502A_F (5'-ggg tga ttt gag caa aat ggc aga att agc ata cgg taa aat acc tg-3') and ClpB_Q502A_R (5'-cag gta ttt tac cgt atg cta att ctg cca ttt tgc tca aat cac cc-3') |
| pKK289-clpB_Y503A_ | ClpB_Y503A_F (5’- cta gct cag gta ttt tac cgg ctt gta att ctg cca ttt tgc tca aat cac -3') and ClpB_Y503A_R (5’-gtg att tga gca aaa tgg cag aat tac aag ccg gta aaa tac ctg agc tag -3’) |
| pKK289-clpB_E508A_ | ClpB_E508A_F (5'-gaa tta caa tac ggt aaa ata cct gcg cta gaa gca caa att aaa caa ata-3') and ClpB_E508A_R (5'-tat ttg ttt aat ttg tgc ttc tag cgc agg tat ttt acc gta ttg taa ttc-3') |
| pKK289-clpB_E510A_ | ClpB_E510A_R (5'-ctt cta ttt gtt taa ttt gtg ctg cta gct cag gta ttt tac cgt at-3') and ClpB_E510A_F (5'-ata cgg taa aat acc tga gct agc agc aca aat taa aca aat aga ag-3') |
| pKK289-clpB_EC_ | E. coli ClpB_5NdeI (5'-GGG TTT CAT ATG CGT CTG GAT CGT CTT ACT A-3') and E. coli ClpB_3SacI (5'-CG ACA GAG CTC TTA CTG GAC GGC GAC AAT CC-3') |
| pKK289-ΔNclpB | U112ClpB_del_1-156_F (5'-gtg tat tta tct aat gca cct ttc att att tcc cct caa gaa aaa tta tct gtt a-3') and U112ClpB_del_1-156_R (5'-taa cag ata att ttt ctt gag ggg aaa taa tga aag gtg cat tag ata aat aca c-3') |
| U112-clpB substitution mutants (*in cis*) |  |
| ClpB_K212A_  (WA1) | ClpB K212A_F (5’- Ggt gag cct ggt gtt ggt gca act gct att gtc gag gg-3’) and ClpB K212A_R ( 5’-Ccc tcg aca ata gca gtt gca cca aca cca ggc tca cc-3’); |
| ClpB_K613A_  (WA2) | ClpB K613A_F (5’-Agg tcc aac tgg tgt cgg tgc aac tga gct tac aaa agc t-3’) and ClpB K613A_R ( 5’-Agc ttt tgt aag ctc agt tgc acc gac acc agt tgg acc t-3’) |
| ClpB_K212A/K613A_  (WA12) | ClpB K212A_F, ClpB K212A_R, ClpB K613A_F and ClpB K613A_R |
| ClpB_E279A_  (WB1) | ClpB E279A_F (5’- Gta atg taa ttc tct tta tag atg cat tac ata cta tgg tag gtg ctg g-3’) and ClpB E279A_R (5’- Cca gca cct acc ata gta tgt aat gca tct ata aag aga att aca tta c-3’); |
| ClpB_E680A_  (WB2) | ClpB E680A_F (5’- Gca tga gct ttt tca acc gcg tct agc aag att aca g-3’) and ClpB E680A_R (5’- Ctg taa tct tgc tag acg cgg ttg aaa aag ctc atg c-3’) |
| ClpB_E279A/E680A_  (WB12) | ClpB E279A_F, ClpB E279A_R, ClpB E680A_F and ClpB E680A_R |
| clpB_R332A_  (Arg1) | ClpB_R332A_F (5’- Gaa aga tcc agc tct aga gag agc att cca aaa agt gct agt tga t-3’) and ClpB_R332A_R (5’- Atc aac tag cac ttt ttg gaa tgc tct ctc tag agc tgg atc ttt c-3’) |
| clpB_R757A_  (Arg2) | ClpB_R757A_F (5’- Caa ttg cat cat caa ctg cat tta caa att cag gtc taa agt ggc taa gta c-3’) and ClpB_R757A_R (5’- Gta ctt agc cac ttt aga cct gaa ttt gta aat gca gtt gat gat gca att g-3’) |
| ClpB_R332A/R757A_ (Arg12) | ClpB R332A_F, ClpB R332A_R, ClpB R757A_F and ClpB R757A_R |

^a^ Underlining in the primer set indicates the incorporated restriction endonucleases sites, as indicated with the primer labels, used for cloning of the PCR-amplified DNA fragments. To generate the *in-cis* complementation primers, a web-based QuikChange Primer Design Program ([www.agilent.com/genomics/qcpd](http://www.agilent.com/genomics/qcpd)) was used.
