# Supplementary material for: Dissociation between the critical role of ClpB of Francisella tularensis for the heat shock response and the DnaK interaction and its important role for efficient type VI secretion and bacterial virulence
Source: PLoS Pathog. 2020 Apr 10;16(4):e1008466. doi: 10.1371/journal.ppat.1008466 (PMC7182274; doi:10.1371/journal.ppat.1008466)
Supplement: S3 Table — (DOCX) [file ppat.1008466.s010.docx]

**S3 Table. Molecular Dynamic Simulation (MDS), 100ns**

| Donor | Acceptor | Occupancy |
| --- | --- | --- |
| GLN502-Side | GLN52-Main | 0.05% |
| ARG56-Side | GLN502-Side | 5.28% |
| GLN262-Side | TYR503-Main | 7.89% |
| LYS497-Side | ASN284-Main | 0.90% |
| ARG56-Side | GLU510-Side | 42.84% |
| LYS270-Side | SER496-Side | 0.62% |
| ARG263-Side | GLU500-Side | 42.46% |
| LYS258-Side | GLU508-Side | 24.54% |
| GLN502-Side | GLN52-Side | 0.05% |
| LYS514-Side | ASP44-Side | 0.43% |
| GLN52-Side | GLN502-Side | 0.52% |
| TYR503-Side | GLN262-Side | 0.48% |
| ARG265-Side | TYR503-Side | 0.05% |
| TYR503-Side | GLN262-Main | 0.10% |
| SER496-Side | GLU266-Side | 12.46% |
| ARG56-Side | GLN502-Main | 0.52% |
| TYR503-Side | GLU269-Side | 23.35% |
| GLN57-Side | GLU508-Side | 13.22% |
| LYS258-Side | GLN512-Side | 0.14% |
| LYS514-Side | VAL59-Main | 0.81% |
| LYS514-Side | THR60-Main | 2.95% |
| TYR289-Side | GLU105-Side | 25.58% |
| HIE297-Main | ASN100-Main | 2.04% |
| HIE297-Side | ASP104-Main | 17.69% |
| ASN284-Side | ASN100-Side | 4.04% |
| TYR287-Side | GLY103-Main | 5.71% |
| ASN100-Side | ASN284-Side | 9.42% |
| ASN299-Side | GLU97-Side | 1.90% |
| GLN96-Side | ASN284-Side | 0.14% |
| LYS514-Side | ASN61-Side | 1.81% |
| LYS107-Side | GLU518-Side | 0.05% |
| GLN96-Side | ASN299-Side | 0.05% |
| LYS527-Side | GLU108-Side | 4.90% |
| LYS296-Side | LYS101-Main | 0.24% |
| LYS527-Side | ASP44-Side | 3.52% |
| LYS296-Side | ASN102-Main | 0.43% |
| LYS55-Side | TYR503-Side | 0.05% |
| LYS107-Side | GLU522-Side | 0.10% |
| GLN490-Side | GLU31-Main | 0.05% |
| ARG530-Side | ASP44-Side | 0.10% |
| ASN100-Side | ASN299-Side | 0.33% |
| THR60-Side | GLU510-Side | 8.80% |
| LYS514-Side | ASP63-Side | 0.10% |
| ASN284-Side | GLN96-Side | 2.00% |
| LYS527-Side | LYS107-Main | 0.05% |
| LYS258-Side | GLY504-Main | 0.05% |

Blue: Amino acids of the ClpB

Red: Amino acids of the DnaK
