# Supplementary material for: Dissociation between the critical role of ClpB of Francisella tularensis for the heat shock response and the DnaK interaction and its important role for efficient type VI secretion and bacterial virulence
Source: PLoS Pathog. 2020 Apr 10;16(4):e1008466. doi: 10.1371/journal.ppat.1008466 (PMC7182274; doi:10.1371/journal.ppat.1008466)
Supplement: S4 Table — (DOCX) [file ppat.1008466.s011.docx]

**S4 Table. Predicted change in protein stability upon introduction of point mutations**

| Methods | | Site Directed Mutator (SDM) | | | Multi agent stability prediction (MAESTRO^3^) | |
| --- | --- | --- | --- | --- | --- | --- |
| No | Mutations | SS^1^ Wild  /Mutant | OSP^2^ values  Wild/Mutant | Predicted  ΔΔG | Predicted  ΔΔG | CPred^4^ |
| 1 | K212A | H/H | 0.43/0.39 | -0.64 | 0.075 | 0.906 |
| 2 | E279A | b/b | 0.18/0.25 | 0.23 | 0.186 | 0.898 |
| 3 | R332A | g/g | 0.22/0.30 | -0.12 | 0.186 | 0.898 |
| 4 | K613A | H/H | 0.46/0.42 | -1.05 | 0.182 | 0.907 |
| 5 | E680A | l/l | 0.36/0.40 | 0.22 | 0.209 | 0.882 |
| 6 | R757A | a/a | 0.34/0.41 | 0.05 | 0.098 | 0.904 |
| 7 | K212A/K613A | - | - | - | 0.116 | 0.913 |
| 8 | E279A/E680A | - | - | - | 0.176 | 0.872 |
| 9 | R332A/R757A | - | - | - | 0.143 | 0.903 |

^1^OSP: Occlude surface packing

^2^SS: Secondary structure. Highly stabilising (G > 2.5 kcal/mol) and destabilizing mutations (G > –2.5 kcal/mol). Highly destabilising mutations (OSP > 0.56).

^3^MAESTRO: stability (kcal/mol). ΔΔGpred.<0.0.

^4^Cpred is a confidence estimation, 0.0 (not reliable) and 1.0 (highly reliable).
