# Supplementary material for: Dissociation between the critical role of ClpB of Francisella tularensis for the heat shock response and the DnaK interaction and its important role for efficient type VI secretion and bacterial virulence
Source: PLoS Pathog. 2020 Apr 10;16(4):e1008466. doi: 10.1371/journal.ppat.1008466 (PMC7182274; doi:10.1371/journal.ppat.1008466)
Supplement: Attachment — Submitted filename: Reply reviewers PlosP 200303-AS.docx [file ppat.1008466.s013.docx]

Dear Dr Eshragi,

Thank you for the review of our manuscript PPATHOGENS-D-19-01936 ” Dissociation between the critical role of ClpB of *Francisella tularensis* for the heat shock response and the DnaK interaction and its important role for efficient type VI secretion and bacterial virulence". We appreciate the constructive comments and believe that they contribute to improve the manuscript. Therefore, we have modified the manuscript accordingly. The changes in the manuscript are marked in yellow.

Please find our point-by-point reply below.

Sincerely,

Anders Sjöstedt

Reviewer #1: (No Response)

Reviewer #2:

1. The biochemical characterization of the ClpB mutants is in parts incomplete. The authors need to document the oligomerization behavior of the variants, as hexamer formation is critical for ClpB function. This has not been done for ATPase mutants and is only mentioned as “data not shown” (page 8, line 166) for M-domain mutants.

As suggested, we have now performed analysis of the conformation of the generated mutants and their oligomerization patterns have been determined. The results have been presented as an additional figure (S2 Fig) and a paragraph has been added in the Results section.

Similarly, the authors did not determine ATPase activities of all ClpB mutants. Most mutant proteins have been purified and therefore completion of the biochemical characterization seems doable.

As suggested, the ATPase activity of the generated mutants has now been determined and the results summarized in Fig. 2D and Fig, 4E and two paragraphs have been added in the Results section.

2. Figure 5D: The defect of F. tularensis ΔclpB cells in intracellular replication can be rescued by plasmid-encoded ΔNTD-ClpB. This result is surprising and is in contradiction to the crucial function of the ClpB NTD in T6SS and F. tularensis proliferation in mice (Fig. 5C/E) but also to Fig. 3A/4A, which document a crucial function of ClpB in the very same assay. The authors need to comment on this unexpected result.

We have now elaborated on this finding and added several sentences in the Discussions to provide an explanation (page 18, line 428-431). We have mentioned that although the finding was surprising, it is not unprecedented, since a few T6SS component mutants previously have been demonstrated to display normal intra-macrophage replication but strong attenuation in an animal model. As concluded in the Discussion; the findings illustrate that the requirements of full virulence *in vivo* are more complex than those of intra-macrophage replication.

3. The ClpV NTD harbors an additional α-helix that is crucial for binding to the T6SS substrate. This helix is missing in ClpB, suggesting that the interaction details of ClpV and ClpB with VipB/IglB substrates are different. The authors shortly report on this aspect in the discussion section (page 16, lines 365-367), but a more detailed analysis and comparison of the substrates (IglB: ClpB; VipB: ClpV) is required here. It is recommended to at least show sequence alignments of IglB and VipB homologs and to highlight the differences in their N-terminal regions (which in case of VipB includes the binding site for ClpV).

We believe that this information will be important to the manuscript and therefore an additional figure has been added, Fig. 5, illustrating the sequence similarities between IglB and VipB as well as their structural similarities.

4. Figure 6: The authors show that the yeast ClpB homolog, Hsp104, cannot function in F. tularensis T6SS. The molecular reason remains unclear as Hsp104 shares substantial sequence homology with ClpB including the NTD. Furthermore, the authors did not determine Hsp104 expression levels, leaving the possibility that Hsp104 is inactive due to poor expression. The presented data therefore do not add much to the manuscript and should be removed.

As suggested, the data regarding Hsp104 have been removed.

Reviewer #3: line 110. The authors begin the results section by describing how they looked for potential interactions between ClpB and the DnaK/Hsp70 chaperone system but no background information was provided for the DnaK/Hsp70 chaperone system, which actually is complicated - including DnaK (Hsp70), DnaJ, and GrpE. Please explain. More importantly, it is not clear why DnaK/Hsp70 is being examined in this study, compared to DnaJ or GrpE.

To better elaborate on the role of the other components, DnaJ and GrpE, of the DnaK/Hsp70 chaperone system, we have added information in the Introduction (page 5, line 85-91). Also, in the Results section, we have clarified that the focus in the manuscript on DnaK is due to its direct interaction with ClpB, whereas DnaJ and GrpE do not directly interact with ClpB (page 6, line 115-117).

line 149-153. The authors note that Q502A showed very most reactivation (19%), while Y503A showed only background activity. However, Figure 2A appears to indicate little difference between KJE alone, Y503A, and Q502A. How can Y503A be background but Y503A has a measurable difference?

In the original submission, we denoted the effect of Q502A as “very modest” and we believe that this accurately reflects the data shown in Fig. 2A. As the reviewer states, there is indeed almost no difference between the activity of Q502A and of Y503A. We have now stated that the effect of Y503A is “….very modest change in aggregate turbidity, not different from the background level”

line 162-163. The authors note that the “disaggregation activities of E500A (61%) and E508A (68%) were … not much lower than that of wild-type ClpB (83%).” However, this reviewer believes that 15% and 22% differences are substantial.

As suggested, we have now rephrased the relevant sentence to better reflect the observed differences (page 8, line 169-171).

line 219-220. It is not clear from S1A Fig, Figure 4, or the materials and methods what specifically was done to generate single or double mutants within both NBDs. Supplemental Table S1 provides this information but it is not immediately clear what was done. Please indicate here so readers don’t have to dig through supplemental material.

This information was in the original submission explicitly stated in Materials and Methods (page 28, line 652-657), but as suggested, we have now added information regarding the generation of the mutants in the figure legend (Fig. 4) to clarify the targeted mutagenesis strategy for the reader. For better understanding, also an additional supplementary figure (S4 Fig) has been included.

lines 236-246 and Figure 4C. It is not clear what point the authors are trying to make about higher secretion levels in WA2, WB1, WB2, and Arg2…is this relevant, substantial, and/or significant? The last sentence about structural topology and protein stability of double mutants also is not clear, given ambiguous data in Figure 4C. All of this needs to be clarified.

As suggested, the appropriate sentences have been rephrased (and also shortened) to more clearly reflect the relevance of the presented data (page 12, lines 261-263).

line 247-251. Perhaps the largest flaw of the paper is in this section, where the authors note ‘the inhibition of ATP binding and hydrolysis….” Where is the data specifically showing ATP binding to ClpB and these ClpB mutants? Where is the data specifically measuring ATP hydrolysis?

As also suggested by reviewer 2, we have now added data regarding the ATPase activity of the generated mutants as summarized in Fig. 2D and Fig. 4E.

Totally aside from ATP binding and hydrolysis, why didn’t the authors generate other ClpB mutants (mutating 4 amino acids or 8 amino acids) outside of the NBD domain and test these mutants in these same assays? How are the authors sure that these specific regions (WA1, WB1, Arg1, etc.) actually account for defects in shock survival, intracellular replication, and T6 secretion?

We have now added a sentence in the Results section to emphasize that each NBD mutation involves only a substitution of a single amino acid (page 11, line 242-246). Moreover, this information was in the original submission explicitly stated in Materials and Methods page 28, line 652-657) and this part has now been extended to further clarify. There are several examples in the manuscript of the same type of single amino acid substitutions, e.g., E510A, without any phenotypic effect, thereby serving as a negative control for the very significant effects of the NBD mutations. To further emphasize this fact, it was also mentioned in the Discussion (page 17, lines 383-385) and a supplementary figure (S4 Fig) has been added.

line 274-283. Given the substantial amount of modeling and domain predictions in other parts of the paper (S1A and Fig 5), it is surprising that the authors decided to delete the N-terminal 156 amino acids of ClpB (i.e. a major percentage of the ClpB protein)

We have, in fact, performed *in silico* modeling of the effects of amino acid substitutions of the N-terminal. However, many mutations are predicted to affect the overall conformation of the N-terminal. Therefore, identification of mutations that affect the phenotype of ClpB, cannot unambiguously be attributed to a critical role for T6S regulation of the mutated amino acid, since it instead may be due to its effect on the tertiary structure of the N-terminal. This analysis therefore requires careful experimental determination of the resulting structures of the mutated forms of the N-terminal and this is technically challenging and time-consuming and therefore outside the scope of the manuscript. On the other hand, our own data (S2 Fig and S5 Fig) and modeling indicated that deletion of the N-terminal did not affect the overall conformation of the protein. This is in agreement with numerous studies regarding the ClpB N-terminal from other bacterial species. Thus, this supports the conclusion that the observed phenotype is due to the function of the N-terminal and not due to effects on conformation.

**Part III – Minor Issues: Editorial and Data Presentation Modifications**
Please use this section for editorial suggestions as well as relatively minor modifications of existing data that would enhance clarity.

Reviewer #1: Line 74 – citation “14” is wrong for this statement. The correct would be: Basler et al., Nature 2012 and Basler & Mekalanos, Science 2012.

The reference has been substituted as suggested.

Line 369 – citation “37” is wrong for this statement (see above).
The reference has been substituted as suggested.

Line 353 - wrong citation.
The sentence has been slightly rephrased to correctly reference the citation used.

Figure 2A,B – show error bars and averages rather than representative data.

The figure has been revised to include error bars and averages as suggested.

Figure 3B - Do authors have a control where a critical component of T6SS was deleted? To know if the observed amount of IglC in the supernatant of ClpB-negative cells appeared there in a T6SS dependent manner.

The figure has been revised to and the suggested control included.

Figure S4 - labels missing.

As suggested, labels have been added.

Reviewer #2: 1. page 7, line 149: In Fig. 2A the authors determine the disaggregation activity of ClpB (wild type and mutants) by monitoring the decrease of Malate Dehydrogenase aggregate turbidity. The term “reactivation” is therefore incorrect for this experimental setting.

To properly reflect what is measured, the term “reactivation” has been substituted with the phrase “disaggregation”.

2. Page 7, line 154: The term “re-aggregation” activity of Y503A is unclear and needs to be defined.

The sentence has now been rephrased and the term “re-aggregation” removed.

3. Suppl. Fig. 3B: It is unclear which data points are depicted here. Do they belong to ClpB wild type and/or mutant proteins?

The data points have been clarified. In addition, the CD and thermal stability data of ΔNClpB have been added.

Reviewer #3: line 109-110: should be “however, the interaction between ClpB and…”

The sentence has been rephrased as proposed.

Fig 5A vs. S1A. Fig 5A shows R503 but no R is present in at position 503 in S1A. Similarly, Fig 5A shows T556 but no T is present at position 556 in S1A. Why doesn’t Fig 5A include relative positions of Arg1 and Arg2?

The designations R503 and T556 are typographical errors and should refer to R530 and T546, as shown in S1A. We are grateful that these mistakes were identified by the reviewer. As suggested, the positions of Arg1 and Arg2 have been illustrated in Fig. 6A (old Fig. 5A).
